# QTL mapping for fruit quality in *Citrus* using DArTseq markers

**DOI:** 10.1186/s12864-017-3629-2

**Published:** 2017-04-12

**Authors:** Maiara Curtolo, Mariângela Cristofani-Yaly, Rodrigo Gazaffi, Marco Aurélio Takita, Antonio Figueira, Marcos Antonio Machado

**Affiliations:** 1 Centro de Energia Nuclear na Agricultura – Universidade de São Paulo (USP), 13400-970 Piracicaba, SP Brazil; 2 Centro APTA Citros Sylvio Moreira – Instituto Agronômico (IAC), CP 04, 13490-970, Cordeirópolis, SP Brazil; 3 Universidade Federal de São Carlos (UFSCar) – Centro de Ciências Agrárias, Rodovia Anhanguera, km 174, 13600-970 Araras, SP Brazil

**Keywords:** Integrated linkage map, Synteny, Sweet orange, Mandarins, Hybrids

## Abstract

**Background:**

Citrus breeding programs have many limitations associated with the species biology and physiology, requiring the incorporation of new biotechnological tools to provide new breeding possibilities. Diversity Arrays Technology (DArT) markers, combined with next-generation sequencing, have wide applicability in the construction of high-resolution genetic maps and in quantitative trait locus (QTL) mapping. This study aimed to construct an integrated genetic map using full-sib progeny derived from Murcott tangor and Pera sweet orange and DArTseq™ molecular markers and to perform QTL mapping of twelve fruit quality traits. A controlled Murcott x Pera crossing was conducted at the Citrus Germplasm Repository at the Sylvio Moreira Citrus Centre of the Agronomic Institute (IAC) located in Cordeirópolis, SP, in 1997. In 2012, 278 F_1_ individuals out of a family of 312 confirmed hybrid individuals were analyzed for fruit traits and genotyped using the DArTseq markers. Using OneMap software to obtain the integrated genetic map, we considered only the DArT loci that showed no segregation deviation. The likelihood ratio and the genomic information from the available *Citrus sinensis* L. Osbeck genome were used to determine the linkage groups (LGs).

**Results:**

The resulting integrated map contained 661 markers in 13 LGs, with a genomic coverage of 2,774 cM and a mean density of 0.23 markers/cM. The groups were assigned to the nine Citrus haploid chromosomes; however, some of the chromosomes were represented by two LGs due the lack of information for a single integration, as in cases where markers segregated in a 3:1 fashion. A total of 19 QTLs were identified through composite interval mapping (CIM) of the 12 analyzed fruit characteristics: fruit diameter (cm), height (cm), height/diameter ratio, weight (g), rind thickness (cm), segments per fruit, total soluble solids (TSS, %), total titratable acidity (TTA, %), juice content (%), number of seeds, TSS/TTA ratio and number of fruits per box. The genomic sequence (pseudochromosomes) of *C. sinensis* was compared to the genetic map, and synteny was clearly identified. Further analysis of the map regions with the highest LOD scores enabled the identification of putative genes that could be associated with the fruit quality characteristics.

**Conclusion:**

An integrated linkage map of Murcott tangor and Pera sweet orange using DArTseq™ molecular markers was established and it was useful to perform QTL mapping of twelve fruit quality traits. The next generation sequences data allowed the comparison between the linkage map and the genomic sequence (pseudochromosomes) of *C. sinensis* and the identification of genes that may be responsible for phenotypic traits in *Citrus*. The obtained linkage map was used to assign sequences that had not been previously assigned to a position in the reference genome.

**Electronic supplementary material:**

The online version of this article (doi:10.1186/s12864-017-3629-2) contains supplementary material, which is available to authorized users.

## Background

Sweet orange (*Citrus sinensis* L. Osbeck) is one of the most commonly consumed fruits in the world, whether in the form of fresh fruit, concentrated and frozen juice (FCOJ) or pasteurized juice (NFC). In Brazil, 18 million tons of oranges are harvested each year, representing 35% of global fruit production and 56% of juice production [1].

In general, the *Citrus* genus has biological characteristics that contribute to an extremely long and challenging breeding process. Because the species are vegetatively propagated, the cultivars of great agronomic interest typically have a high number of heterozygous loci, making the development of varieties from crosses difficult due to the segregation observed in the progeny, including for those traits with strict varietal standards. In addition, the polyembryonic nature, the presence of sterile eggs and pollen grains, and the existence of gametophytic incompatibility in some varieties represent additional genetic barriers. As perennial tree species, citrus requires a long juvenile period before blossoming and bearing fruits [2–5]. Because of these genetic and botanical obstacles, the vast majority of existing varieties today originated from the selection of spontaneous mutants carrying desirable characteristics. The objective of traditional programs of citrus breeding is to obtain scion and rootstock that carry resistance to diseases and pests, are more adapted to adverse abiotic conditions and produce standard high-quality fruits. In this context, molecular markers can be useful for crop improvement since they detect existing variations in the genome and allow access to information about the genetic control of important features such as disease resistance, fruit quality attributes and tolerance of abiotic stress, thus reducing the time required for obtaining superior new varieties.

Genetic maps are useful tools for identifying genetic polymorphisms in species and elucidating the genetic architecture of quantitative traits. For citrus, 26 genetic maps have been developed in the last 20 years by several research groups, using various types of molecular markers, such as RAPD [6–10], RFLP, AFLP [7, 11, 12], SSR [13–16] and SNP [17]. However, these genetic maps were developed with few markers and resulted in low saturation, which impairs the detection of genomic regions that control characteristics of agronomic interest.

The microarray-based DArT technology, proposed more than 15 years ago, offered the development of a large number of polymorphic markers at a low cost per data point, which favored higher genomic coverage [18]. A new variant, called DArTseq, was later developed using combinations of restriction enzyme digestions to reduce genome complexity, followed by next-generation sequencing to identify DNA polymorphisms [19]; this technology overcame the difficulties related to the low number of markers. The high-throughput capability allowed rapid characterization, sequence data independence, detection of single base changes and indels, and whole genome coverage, in addition to providing the sequence obtained from each marker [20]. This technology has enabled the application of DArTseq markers for the analysis of genetic population variability, high-density genetic mapping and genomic assembly support.

A complete genome represents a valuable resource for understanding many important citrus traits, and a consolidated map can aid in genome assembly and increase the resolution. The *C. sinensis* sweet orange contains nine pairs of chromosomes and has an estimated genome size of 367 Mb. In the genome version proposed by Xu et al. [21], the contig sequence length covers 87.3% of the estimated sweet orange genome, and more than 80% of the genome assembly is in 135 scaffolds, with 239 Mb anchored in nine linkage groups (LGs); however, in the citrus genome, 15% of the contigs are unassigned in the nine reference chromosomes [21]. These sequences exhibited fragmented assembly to the genome scaffolds and were subsequently reported as belonging to chromosome Un (unassigned). Therefore, improving the assemblies and annotations remains a work in progress. Experimental approaches have aimed to improve the connectivity of the contigs and scaffolds, assigning and ordering scaffolds on the chromosomes.

Using an integrated linkage map obtained from DArTseq markers, the present study aimed to detect and characterize the quantitative trait locus (QTL) associated with fruit quality traits in an F_1_ progeny obtained from a controlled cross of Murcott (TM) x Pera sweet orange (LP). Further, the study aimed to identify the gene content at intervals in the QTL, as detected by comparative analysis between the integrated linkage map and the *C. sinensis* reference genome. The contributions of the available markers with known sequences and the sequenced citrus genomes, as well as the generation of additional information from the genomic assembly, will be discussed.

## Methods

### Mapping population

A controlled cross between a Murcott tangor (female parent) and a Pera sweet orange (pollen donor) was conducted in the spring of 1997. The identification of individual zygotic plants was conducted using morphologic, RAPD and SSR markers; approximately 2,000 seedlings were obtained, but a total of 312 hybrids were verified as zygotic [22]. From these hybrids, 278 plants were randomly selected to establish the population for the mapping study.

The hybrids and parents were grafted onto Rangpur lime (*C. limonia* Osbeck). The experiment to evaluate the fruit quality characteristics was established in 2006 at the Sylvio Moreira Citrus Center of the Agronomic Institute using a completely randomized design, with three replicates of each hybrid. The fruit characteristics were assessed in July 2012.

### DNA Extraction and molecular marker analysis

Total DNA was extracted from fresh leaves according to Machado et al. [23]. DArTseq genotyping was conducted using *Pst*I and *Taq*I digestions, and sequencing was performed with a HiSeq 2000 sequencing system (Illumina Inc., San Diego, USA) at Diversity Arrays Technology Pty Ltd., Australia. The resulting sequences were filtered for quality, with a cut-off at 90% confidence. Sequences were aligned with the Clementine tangerine reference genome (www.phytozome.org). Using this procedure, approximately 30,000 markers were obtained. The generated DArTseq markers were coded as “0” or “1”, according to their absence or presence, respectively. All markers were analyzed as hybrids according to the Mendelian segregation patterns that are expected for F_1_ progeny.

### Linkage Map

For the construction of the integrated genetic map, all DArTseq loci that showed no deviation from the expected segregation of 1:1 [heterozygous for the male parent (oo x ao) and the female parent (ao x oo)] and 3:1 (ao x ao) were considered. The selected markers were coded according to Wu et al. [24]. OneMap software [25], which is based on a multipoint approach using hidden Markov models, was used with the following settings: the likelihood ratio was used to form the LGs considering an LOD score = 8 and a maximum recombination fraction of 0.3. The genomic information obtained from the sequenced *C. sinensis* genome, available at http://citrus.hzau.edu.cn/orange/index.php, was also considered when declaring the LGs.

After the formation of the groups, a preliminary marker ordering using the RCD (rapid chain delineation; Doerge [26]) algorithm was established to remove redundant markers, i.e., markers showing a 0.0 recombination fraction. In these cases, only one of the markers was maintained in the map. Next, we ordered the markers within each group. However, for the LGs with a reduced number of 3:1 markers (C, heterozygous for the both parents – ao x ao, fewer than 10), an integration between the 1:1 markers (D1, heterozygous for the tangor Murcott parent - ao x oo) and 1:1 (D2, heterozygous for the Pera parent - oo x ao) markers would not be efficient. In this case, we obtained two groups: one group with markers from 1:1 (polymorphic for Murcott tangor) and 3:1, coded as “a,” and a second group with markers from 1:1 (Pera) and 3:1, designated “b.” Briefly, each group was ordered using an exhaustive search to sample a set of markers containing genomic information; the best order was achieved by choosing the marker with the highest likelihood value using the COMPARE command. Other markers were inserted in the LGs using the TRY command, according to the most likely position. The distances on the map or the centiMorgan (cM) linkage between the different loci were estimated using the Kosambi mapping function [27] with the recombination frequency conversion.

### Phenotypic trait analyses-characterization of fruits

These analyses were performed in the Quality and Postharvest Laboratory at the Sylvio Moreira Citrus Center of the Agronomic Institute. The number of seeds and segments per fruit was evaluated after cutting the fruit in half and performing a direct count. The ease of peeling was evaluated by three evaluators using a scale from 1 (hard) to 2 (moderate) to 3 (easy). The results represent the average measurement of 3 fruits. The fruits were physically and chemically evaluated using a sample from each repetition. Therefore, five fruits were used for the following evaluations: the fruit mass was determined on a Filizola® balance with a capacity of 15 kg and an accuracy of 5 g and the longitudinal (‘height’) and transversal (‘diameter’) diameters of the fruits were estimated using a graduated ruler. The number of fruits/box was obtained by dividing 40.8 kg (capacity of the box) by the average weight of the fruit. The fruit juice yield was calculated according to the mass ratio of the juice/fruit mass after the fruit had been crushed on an OIC OTTO 1800 juice extractor; the total soluble solids (TSS) content (Brix) was determined from a direct reading on a refractometer; and the total titratable acidity (TTA) was estimated from the titration of 25 mL of juice with a 0.3125 N NaOH solution, using phenolphthalein as an indicator. The TSS/TTA ratio, which indicates the fruit ripening stage, was calculated. The technological index (TI) was obtained from the following formula: TI = juice yield (%) x TSS (Brix) x weight of the citrus industry standard box (40.8 kg)/10,000, as proposed by Di Giorgi et al. [28].

### Statistical analysis

The adjusted average was calculated for all traits, and the predictions of the genotypic values (BLUE) were used to perform the QTL mapping. The genetic correlations (r_g_) between each pair of traits were obtained using Pearson’s correlation coefficient with the individual genotypic values. These correlations were tested while assuming a global significance level of 0.05. The analyses were performed using R software [29].

### QTL Mapping

We adopted the approach described by Gazaffi et al. [30] for QTL mapping, which consists of composite interval mapping (CIM) [31] under an integrated genetic map. To apply the CIM model, a co-factor selection step was required to control the QTLs located outside of the mapping range. This step was performed using step-wise regression, with the Akaike information criterion and the window size defined as 1,000. To declare a QTL, a significance level of 0.95 and 1,000 permutations were adopted to obtain the threshold [32], according to the modification proposed by Chen and Storey [15].

### Comparative analysis of the integrated linkage map and the *C. sinensis* reference genome

Comparative analysis was performed between the integrated linkage map constructed in this study and the *C. sinensis* reference genome. For this comparison, all marker sequences anchored in the integrated map (Additional file 1: Table S1) were aligned with the genome using the BLASTN tool (http://citrus.hzau.edu.cn/orange/index.php), and only the best result, based on the score, of the first alignment was used for comparison. To facilitate the visualization of results and to enable synteny identification, a graphical display was generated in the Circos table viewer platform (http://mkweb.bcgsc.ca/tableviewer/). The marker order in the linkage map, which represents the physical position of the markers in the genome, was estimated by aligning the nine LGs with sweet orange pseudochromosomes.

### Gene content in the Intervals between the detected QTLs

To analyze the gene content in the intervals between the detected QTLs, the markers that flanked each identified region were aligned with the sequences of the *C. sinensis* genome, and their positions were determined. These positions were considered coordinates to enable searching for the genes located within each interval and their functions (http://citrus.hzau.edu.cn/orange/).

## Results and Discussion

### Marker polymorphism and segregation analyses

In this work, 27,960 DArTseq markers were generated considering parameters of quality. However, it was necessary to discard 10,596 markers, because parental genotype was not revealed by the technology of DartseqTM and consequently the segregation could not be inferred. From 17,364 markers, the χ2 test was performed to determine the segregation distortions, in this case 43% (7,425) of the evaluated markers showed deviations of the expected proportions (1:1 and 3:1), for this reason they were removed from the linkage mapping analysis. It should be noted that, this number is in accordance with what has been validated in other works, for example Oliveira et al. [7] found 61 and 48% of markers with segregation distortion considering significant level of 5 and 1%, respectively, in Pera. Gulsen et al. [8] and Cai et al. [33] have found segregation distortion as 57 and 40%, respectively at significant level of 5%. Some possible hypotheses for the occurrence of segregation distortion in citrus were exposed by Oliveira et al. [12], among which, we can highlight the presence of lethal or sublethal genes, non-random segregation, sampling error and/or the small size of the available population and others. In this study, the use of 278 individuals was advantageous, because it minimized the effect of random sampling, since most of the studies used less than 100 individuals [6, 7, 9–14, 34–37]. Still, the amount of missing data was not high, i.e., among the 17,364 markers, the average of missing data was 7.7%. Therefore, considering these two factors, we believed that the errors from sampling were minimized. Also, a factor that may explain this segregation distortion cited by Oliveira et al. [12], by Ruiz et al. [14] and Song et al. [34] is the presence of recessive lethal factors, which favor some alleles in gametic selection or embryo abortion [14, 34], in this case the markers linked in same region of these loci can have can have distortion in their frequencies by an indirect selection generated by these factors.

On the other hand, 9,939 DArTseq markers showed no segregation deviations and were used in a preliminary analysis to verify redundant markers by constructing the LGs and sorting each group using the RCD algorithm [26]. Thus, it was possible to remove all markers with a 0.0 recombination fraction, and the remaining 932 DArTseq were used to construct the genetic map. One possible explanation for the high number of adjacent markers with a recombination frequency of 0.0 is the restriction enzymes used to develop the DArTseq markers, that could have detected very close regions, but further studies are needed to draw any conclusions. Another possible biological hypothesis for several markers showed recombination fraction 0.0 was sample size used in the mapping procedure. We consider the number of individuals in this study is relatively large if compared with others studies that used 164 [8], 151 [16], 143 [35], 97 [36], 94 [7], 87 [12], 80 [14, 37], 72 [11], 60 [38], 65 [6, 39, 40], 57 [13] and 52 [10] individuals. However, it should be noted the number of individuals used (278) here may not be large enough for dealing with high-output system. In this case, if markers are tight linked blocks of non-recombinant loci are observed. For this, the best solution is the increase of sampling size, in order to verify more recombinant events between loci. If hypothetically higher population size could be considered, block of non-recombinant could be broken. It must be stressed that a large sample size, for example 700 individuals use by Laborda et al., [41] working with *Drosophila mediopunctata* in an F_2_ population, is very complicating or impracticable for linkage studies in citrus genus, especially if QTL mapping studies would be the main goal, as extremely large field trails must be done. We consider that our map is good representation of genome due to all linkage groups were represented and no gaps were verified integrating different segregation pattern. In future studies, larger population could be design to try to maximize the information of DArT technology.

The high-throughput technology of genotyping made it possible to easily obtain a large number of markers in the progeny; however, increasing the size of the population used became problematic. For citrus, this difficulty is even greater because of the need for a large experimental area. The only possible solution for mapping a large number of polymorphisms, especially when only dominant markers are considered, is to increase the number of individuals in the population.

In addition, different regions of the chromosome have different rates of recombination, with some genomic regions called hot spots exhibiting a higher frequency of occurrence. Additionally, the parental genotype may influence the recombination ratio, with intra-specific crosses presenting much more recombination than inter-specific hybrids. Nevertheless, markers with a recombination frequency of 0.0 could be used in association mapping studies.

From the 932 DArTseq markers selected, 160 markers were heterozygous for both parents (3:1 segregation ratio), which accounted for 17.2% of the markers. In addition, several markers showed a 1:1 segregation ratio, where 394 and 378 were heterozygous in the Murcott tangor and in Pera sweet orange parents, respectively. This scenario makes the construction of an integrated linkage map challenging for two reasons: first, a reduced number of markers exhibited a 3:1 segregation ratio, which is essential for integration between the 1:1 segregating marker, and second, markers with a 3:1 segregation ratio are less informative for integration than ratios of 1:2:1 and 1:1:1:1 [24, 39].

### Linkage Map

This was the first study to map DArTseq markers in citrus using the method proposed by Wu et al. [24]. However, due to the absence of two-parent markers in some groups, specific parental LGs were formed when all the markers originated from either the Murcott tangor (a) or the Pera sweet orange (b). From the 932 markers, available to construct the map, 661 were positioned into the LGs.

**Fig. 1:**
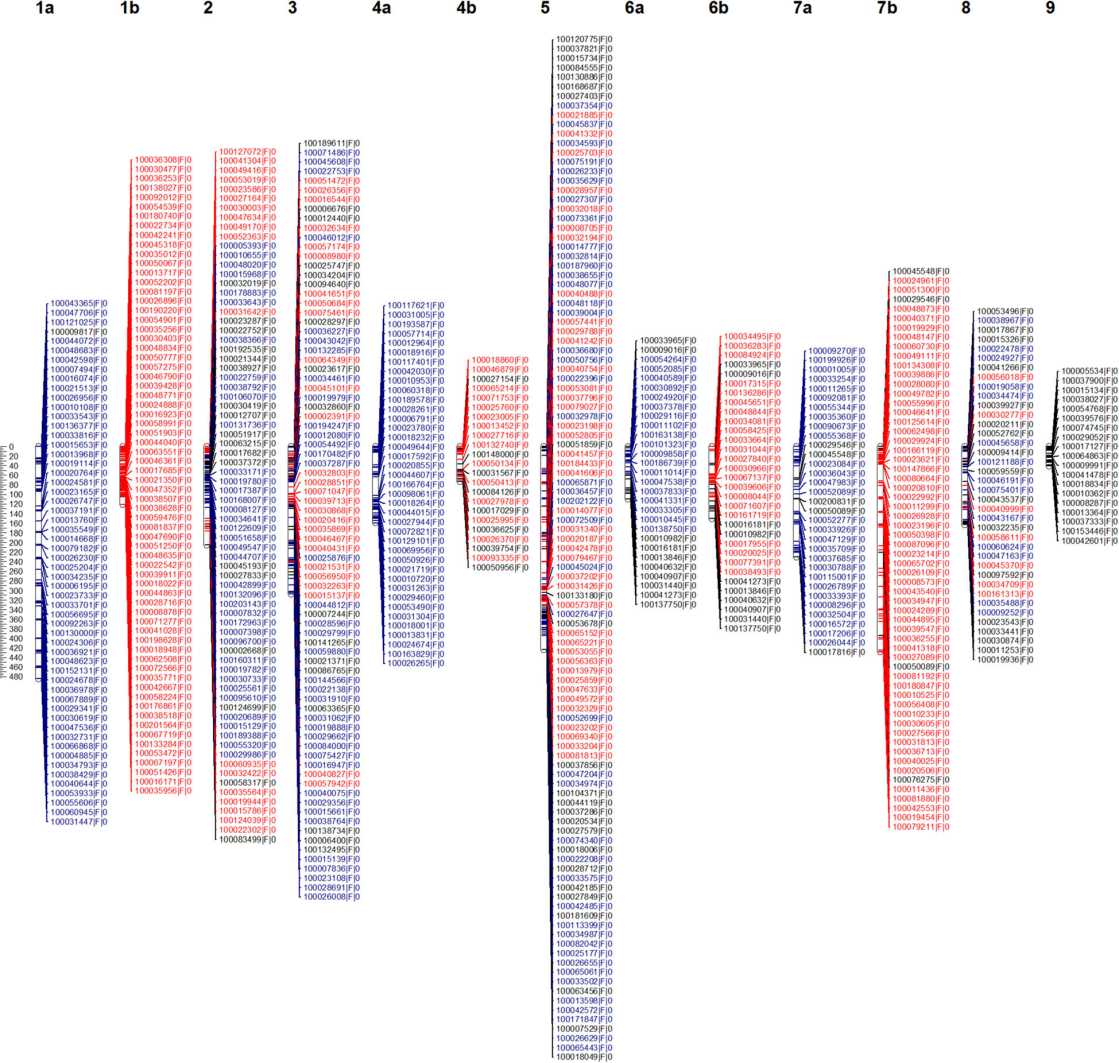
Integrated linkage map constructed in OneMap software. The linkage groups (LGs) marked with “a” and “b” represent the LGs specific for Murcott tangor and Pera sweet *orange*, respectively, being partially integrated. The D1, D2 and C markers are in *blue*, *red* and *black*, respectively

The integrated genetic map has 13 LGs. The size of the groups ranged from 40.48 cM (LG9) to 484.73 cM (LG1a), with a total size of 2,774.29 cM (Fig. 1, Table 1), and the distances between the markers in the LGs ranged from 0.1 to 38.45 cM, with an average distance of 4.19 cM between markers.

**Table 1:** Linkage group, number of markers and size in centiMorgans (cM)

Linkage group	Number of markers DArTseq	Marker C	Marker D1	Marker D2	Size (cM)
Group 1a	56	1	55	0	484.73
Group 1b	68	0	0	68	120.92
Group 2	74	17	38	19	204.77
Group 3	81	17	37	27	307.30
Group 4a	39	0	39	0	159.41
Group 4b	23	2	0	21	72.69
Group 5	109	24	44	41	423.30
Group 6a	29	10	19	0	109.63
Group 6b	32	10	0	22	150.39
Group 7a	33	5	28	0	230.40
Group 7b	60	4	0	56	428.51
Group 8	38	17	14	7	162.98
Group 9	19	19	0	0	40.48
Total	661	109	274	261	2774.29

Such a short distance between the mapped markers reflects the saturation of this map, this is one of the most saturated maps for citrus, once it has a density lower than 5 marker/cM. Ollitrault et al. [17] developed a Clementine reference map (961 markers for 1,084.1 cM). The reference map was established by combining male and female Clementine segregation data using different marker system (Single Nucleotide Polymorphism (SNP), Simple Sequence Repeats (SSR) and Insertion-Deletion (Indel) markers). The authors referred to it as a medium-density map with on average of one marker/cM. Following this concept and comparing with other papers already published by Oliveira et al. [7], Oliveira et al. [12], Raga et al. [16], that documented average distance of 6, 8 and 30 cM between markers, respectively, the integrated map of Murcott tangor and Pera sweet orange can also be considered as a medium density map. The combination of different markers system may help to saturate even more the genetic map published in this work.

When compared this map with the previous map published for Murcott tangor and Pera sweet orange by Oliveira et al. [12], we consider that DArTseq provided good results. These authors reported the construction of two maps, the Murcott map was based in 65 fAFLPs (fluorescent Amplified Fragment Length Polymorphism), average distance between them 29.5 cM, divided into 9 linkage groups (LGs) showing 1,651.47 cM. Pera sweet orange map has 55 fAFLPs, with average distance between them of 31.9 cM, divided into 5 LGs with total size 1596.2 cM. Our map has ten times more markers and it is seven times more saturated.

In the same work proposed by Oliveira et al. [12], a second Murcott map was obtained. But, only markers heterozygous for the Murcott parent (1:1 ratio) and for both parents (3:1 ratio) were considered, resulting in 9 linkage groups with 227 markers, with an average distance of 4.25 cM among them, totalizing 845 cM. Once again, our map can be considered complete and with a better genome coverage, because in our study we have three segregation pattern, i.e., an extra segregation type was included which are markers segregating for Pera parent, which were not considered by Oliveira et al. [12].

Our map contained an average of 0.23 markers/cM. Among the LGs, LG1a and LG1b had the lowest and highest densities of markers, with average distances of 8.6 and 1.7 cM between them, respectively. The size of the citrus genome was estimated to be between 1,500 and 1,700 cM [38]. In this study, the Kosambi function [27] was used to obtain an integrated map with a size of 2,774.29 cM, thus surpassing the other linkage maps obtained for citrus. We believe that the main reason for this map extension is related to the inability to integrate four groups (LG1, LG4, LG6 and LG7) due to the reduced number of markers with a 3:1 ratio compared to those with a 1:1 ratio. Therefore, in our strategy, the best order was obtained by splitting the single group into two groups, performing the integration between the D1 (heterozygous for the tangor Murcott parent - ao x oo) and C (heterozygous for the both parents – ao x ao) markers and the D2 (heterozygous for the Pera parent - oo x ao) and C markers. We recognize that we counted these four groups twice, thus inflating the actual size, but the generated map still provides a higher density than some citrus previous maps. To improve this map, future studies should include co-dominant markers, especially with those segregation ratios of 1:2:1 and 1:1:1:1. SNPs and SSR would probably integrate these four groups, thereby reducing the map length.

The integrated genetic map has 13 LGs reflecting the nine haploid citrus chromosomes. Increasing the coverage of a genetic map, the number of LGs approaches the species haploid number of the chromosomes as the number of unlinked markers approaches zero. Thus, the map obtained in this work represents the haploid number of the chromosomes for the species, with high genomic coverage.

### Phenotypic analyses

**Fig. 2:**
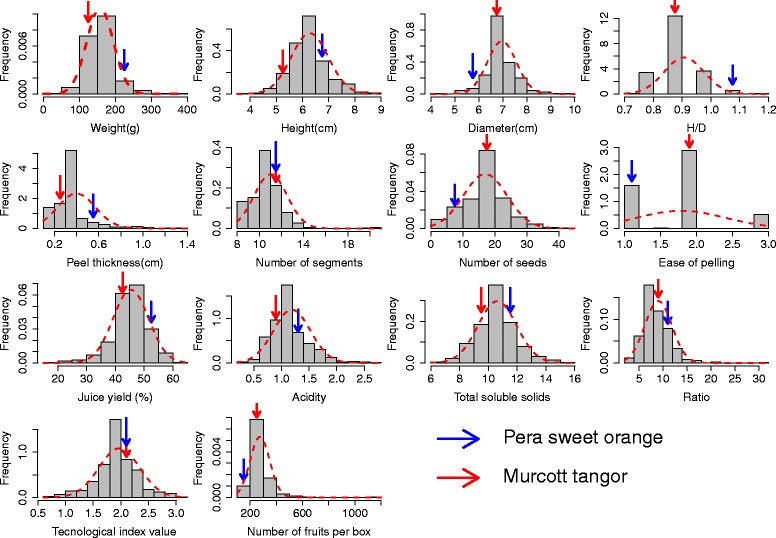
Averages of the frequency distribution for the weight (g), height (cm), width or diameter (cm), height/width ratio (A/D), peel thickness (cm), numbers of buds and seeds, ease of peeling (1 = hard to 3 = easy), juice yield (%), acidity (TTA), total soluble solids (TSS), TSS/TTA ratio, technological index (TI) and number of fruits per box of the segregating population containing 278 individuals; the values of the fruits obtained from Murcott tangor and Pera sweet *orange* parents are indicated by arrows

The mean frequency distribution of the evaluated characteristics was adjusted to a normal distribution (Fig. 2). The maximum, minimum, average and coefficient of variation (CV, %) for the weight, height, diameter, A/D, peel thickness, number of segments, number of seeds, ease of peeling, yield, TTA, TSS, TSS/TTA, TI and number of fruits per box are shown in Table 2.

**Table 2:** Minimum value, maximum value, mean and coefficient of variation (CV, %) of the weight (g), height-A (cm), diameter-D (cm), A/D ratio, peel thickness (cm), number of segments, number of seeds, ease of peeling, juice content (%), total titratable acidity (TTA, %), total soluble solids (TSS), TTS/TTA ratio, technological index (TI) and number of fruits per box in the F1 progeny of Murcott tangor and Pera sweet orange

Trait	Minimum value	Maximum value	Mean	Coefficient of variation (%)
Weight (g)	36.8	353.0	157.4	15.1
Height (cm)	3.5	8.8	6.2	7.7
Diameter (cm)	4.4	9.7	6.9	6.1
A/D	0.7	1.2	0.9	5.6
Peel thickness (cm)	0.1	1.4	0.4	23.9
Number of segments	8.0	21	11.0	10.1
Number of seeds	0.0	44	17.0	26.1
Ease of peeling	1.0	3.0	1.7	7.7
Juice content (%)	19.5	60.1	45.4	8.4
Acidity (%)	0.3	2.8	1.2	17.0
Total soluble solids	6.9	15.9	10.6	8.7
TSS/TTA	2.0	31.0	9.0	19.4
Technological index	0.7	3.2	1.97	12.4
Fruits per box	115	1108	273.8	20.9

In general, the variability of the data (CV) was low at less than 25% for all characteristics (Table 2), except for the number of seeds, which had more heterogeneous data with a CV of 26.1%. Analysis of the field data indicated the high quality of the experiments. Some traits showed evident variability, such as the fruit weight (g), which ranged from 36.8 to 353; the number of segments per fruit, ranging from 8 to 21; and the number of seeds, ranging from 0 to 44 seeds per fruit. Further, the TSS/TTA ratio ranged from 2 to 31, the number of fruits per box ranged from 115 to 1108 (Table 2), and the yield varied from 19.5 to 60.1% among the hybrids.

On the other hand, little variation was observed in other traits among the hybrids. The fruit height ranged from 3.5 to 8.8 cm, whereas the fruit diameter had minimum and maximum values of 4.4 and 9.7 cm, respectively. The ratio between the height/diameter ratio (A/D) showed limited variation from 0.7 to 1.2, and the thickness of the peel ranged from 0.1 to 1.4 cm. Limited variation was found in the fruit acidity, TSS and TI, which ranged from 0.3 to 2.8, 6.9 to 15.9, and 0.7 to 3.2, respectively.

### QTL Mapping

**Fig. 3:**
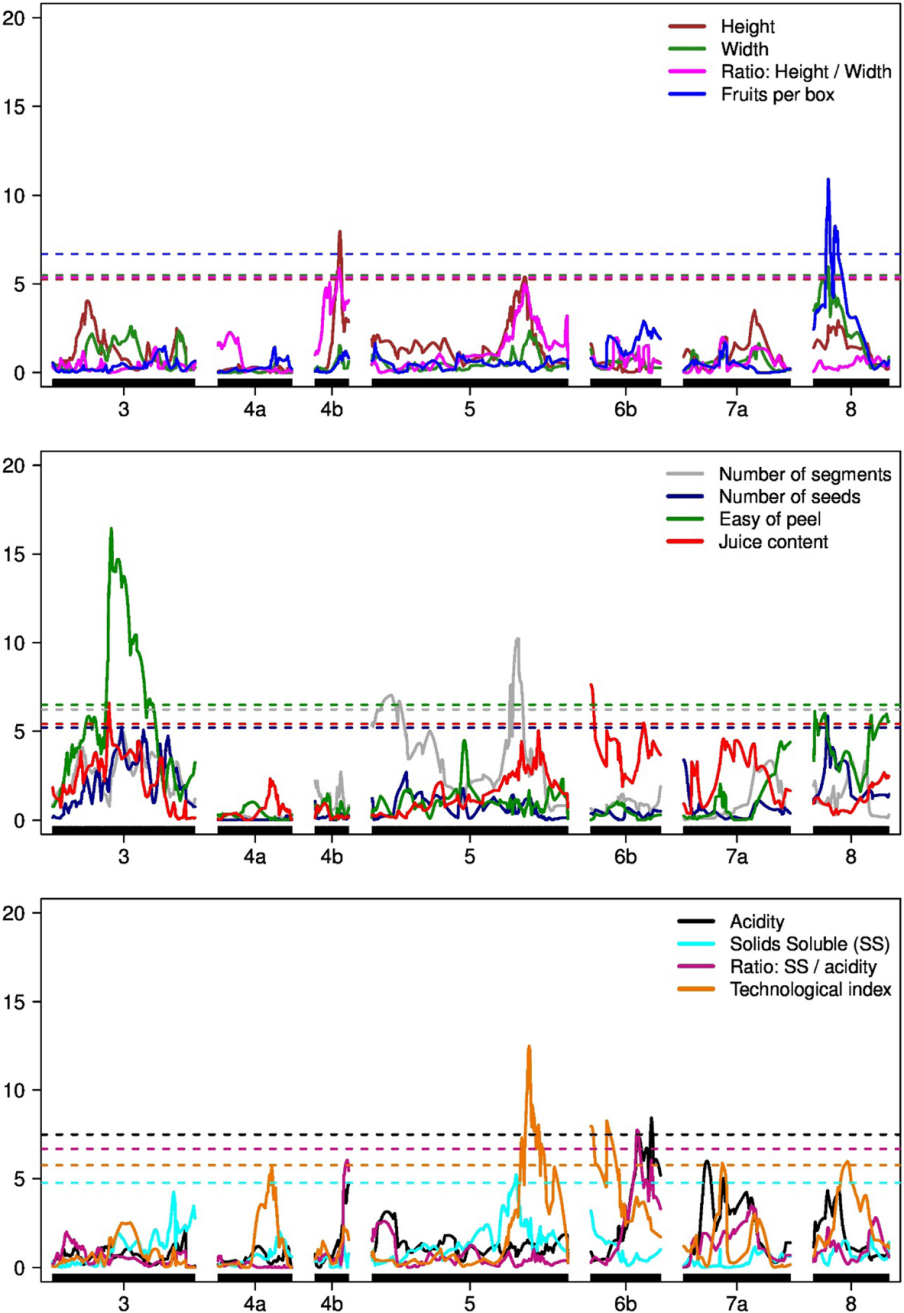
QTL mapping for weight (g), height (cm), width or diameter (cm), height/width ratio (A/D), peel thickness (cm), numbers of buds and seeds, ease of peeling (1 = hard to 3 = easy), juice yield (%), acidity (TTA), total soluble solids (TSS), TSS/TTA ratio, technological index (TI) and number of fruits per box. Note that the dashed lines represent the threshold values obtained with 1000 replicates

QTL mapping was performed for the fruit weight (g), fruit height (cm) fruit diameter (cm), height/diameter ratio, peel thickness (cm), number of segments, number of seeds, ease of peeling, juice content (%), TTA (%), TSS (%), TSS/TTA, TI and number of fruits per box. According to the analysis performed using the CIM method and the threshold obtained from the permutation test, 19 QTLs were detected in 7 LGs for the 12 fruit traits (Fig. 3, Table 4). No QTLs associated with fruit weight or peel thickness were detected, while the fruit TI had the highest number of QTLs (5 QTLs).

In general, the calculated LOD significance thresholds varied from 4.76 (TSS) to 10.77 (peel thickness). However, the LOD score of the detected QTLs ranged from 5.2 to 16.43, indicating consistent regions. The phenotypic variance (R^2^) explained by the markers remained low, ranging from 0.04 to 9.7%, which indicates the polygenic nature of these traits. As most of the markers showed a 1:1 segregation, this was the predominant segregation (7 out of 19) in the detected QTLs. However, other types of QTL segregation, such as 1:2:1, 3:1 and 1:1:1:1, were also detected, which is advantageous for QTL mapping in an integrated genetic map, when a QTL cannot be found using a double pseudo-testcross approach [30, 42].

The fruit height, diameter, height/diameter ratio and number per fruits per box were related with fruit size. Five QTLs were identified for these 4 traits in LG4b (54 cM and 52.23 cM), LG5 (331 cM) and LG8 (32.25 cM). Considering the fruit height, two QTLs were mapped with 1:1 segregation (LG4b at 54 cM), and another QTL was mapped with 1:2:1 segregation (LG5 at 331 cM), thereby explaining 3.58% of the phenotypic variance (R^2^). The first QTL (LG4b) presents one additive effect from Pera, and the other QTL (LG5) has an additive effect from Murcott tangor and a dominance interaction. If only the QTL for the diameter was considered, one QTL was found at LG8 at 32.25 cM, explaining 9.7% of the phenotypic variation, with both parents showing significant additive effects to generate a QTL with a 1∶2:1 segregation pattern. The height/diameter ratio was associated with a single QTL mapped on LG 4b at 52.23 cM, accounting for 3.8% of the phenotypic variation, and the 1∶1 segregation resulted from the significant additive effect from Pera. In addition, one QTL for the number of fruits per box was mapped in LG8 at 32.25 cM, with an R^2^ of 9.7% and a 3:1 segregation pattern because all of its genetic effects were significant.

Notably, the fruit height and height/diameter ratio had a significant correlation of 0.58 (Table 3). This result was corroborated by the QTL mapping because both traits share a common QTL, with the significance peaks separated by only 1.77 cM, and show a 1:1 segregation pattern, with a significant effect from Pera. This effect was not verified by the fruit diameter or the height/diameter ratio, but the correlation was only 0.04 in this case. Most likely, the height/diameter ratio indicates that more variation was observed for the fruit height than the diameter. Another correlation, with a value of−0.10, existed between the number of fruits per box and the fruit diameter, which shared a QTL in LG8 at 32.25, with an additive effect from Murcott tangor and Pera parents. In this model, the signal indicates the linkage phase between the markers and the QTL, and the result can be explained as follows: the allele that increases the diameter is the same allele that reduces the number of fruits per box. Essentially, both traits may carry a pleiotropic QTL, with contrasting effects.

**Table 3:** Pearson correlation values among all traits – weight (g), height-A (cm), diameter-D (cm), A/D ratio, peel thickness (cm), number of segments, number of seeds, ease of peeling, juice content (%), total titratable acidity (TTA, %), total soluble solids (TSS), TSS/TTA ratio, technological index (TI) and number of fruits per box in the F_1_ progeny of Murcott tangor and Pera sweet orange

	Weight	Height	Diameter	A/D	Peel thickness	Segments per fruit	Number of seeds	Ease of peeling	Juice content	Acidity	Soluble solids (TSS)	TSS/TTA	Technological index TI	Fruit per box
Weight	1	0.81**	0.88**	0.19**	0.05	0.05	−0.1*	−0.1*	−0.14**	0.12**	−0.2**	−0.34**	0.07	−0.12**
Height		1	0.76**	0.58**	0.12*	−0.02	−0.11**	−0.11**	−0.11**	0.03	−0.1*	−0.349**	−0.02	−0.19**
Diameter			1	0.04	0	0.06	−0.11**	−0.11**	−0.11**	0.14**	−0.27**	−0.33**	0.13**	−0.1**
A/D				1	0.14**	−0.05	−0.03	−0.03	−0.05	−0.1*	0.12**	−0.15**	−0.16**	−0.16**
Peel thickness					1	−0.02	−0.04	−0.04	−0.17**	−0.21**	−0.04	−0.03	0.01	−0.17**
Segments per fruit						1	0.08*	0.08*	−0.17**	−0.06	−0.09*	−0.08	0.08*	−0.07
Number of seeds							1	0.1*	−0.19**	0.04	0.02	0.03	0.01	0.04
Ease of peeling								1	−0.19**	0.04	0.02	0.03	0.01	0.04
Juice content									1	−0.02	0.01	0.08	0	0.02
Acidity										1	0.03	0.05	0.01	0.73**
Soluble solids											1	0.17**	−0.81**	0.14
TSS/TTA												1	0.2**	0.7**
Technological index													1	0.14**
Fruit per box														1

Note * p < 0.05 and **p < 0.01

Six QTLs for the internal fruit traits (number of segments, number of seeds, ease of peeling and juice content) were mapped to the following LGs: 3 (121.71 cM, 126.49 cM and 148.90 cM), 5 (315,12 cM), 8 (30.42 cM) and 6b (0.0 cM). For the number of segments, only one QTL was mapped at LG5 at 315.12 cM, with an R^2^ value of 1.7% and 3:1 segregation because all of its effects were significant. For ease of peeling, one QTL was detected in LG3 at 126.49 cM, explaining 2.6% of the phenotypic variation, that had a 1:1:1:1 segregation pattern, with significant additive effects that differed from each other (Table 4). For the number of seeds, two QTLs were identified in LG3 at 148.90 cM and in LG8 at 30.42 cM, accounting for 11.1% of the phenotypic variation. The former showed a 3:1 segregation pattern, and the latter showed a 1:2:1 pattern due to a similar additive effect for the Pera parent and a dominance effect. For the juice content, two QTLs were detected: one in LG3 at 121.71 cM with a 1:2:1 segregation pattern and another in LG6b at 0.0 cM with a 1:1 segregation pattern due to a significant additive effect for Pera (Table 4).

**Table 4:** QTLs mapped to the weight (e), height (cm), diameter (in), A/D ratio, number of segments, number of seeds, ease of peeling, juice content, total titratable acidity (TTA), total soluble solids (TSS), TSS/TTA ratio, technological index (TI) and number of fruits per box

Trait	G.L	cM	Marker	LOD	p	LOD	q	LOD	pq	LOD	Seg.	R^2^
Height	4b	54	100084126|F|0–100027978|F|0	7.96	−0.020	0.02	0.25	6.32	−0.06	0.21	1:1	2.0
Height	5	331	100081813|F|0–100037856|F|0	5.38	−0.075	1.23	0.04	0.44	0.13	3.04	1:2:1	1.58
Diameter	8	32.25	100020211|F|0	5.95	0.14	3.78	−0.12	2.97	0.02	0.13	1:2:1	9.7
A/D	4b	52.23	100084126|F|0	5.87	0.02	0.67	0.01	2.41	−0.00	0.14	1:1	3.8
Segments per fruit	5	315.12	100013979|F|0	10.23	0.3	4.16	−0.39	5.9	−0.25	2.8	3:1	1.7
Number of seeds	3	148.90	100059880|F|0	5.23	1.91	3.68	1.90	2.54	1.59	2.06	3:1	1.8
Number of seeds	8	30.42	100039927|F|0	5.86	0.48	0.33	1.36	2.46	−1.21	1.9	1:2:1	9.3
Ease of peeling	3	126.49	100029799|F|0	16.43	−0.18	2.5	0.41	12.1	0.06	0.47	1:1:1:1	2.6
Juice content	3	121.71	100032263|F|0	6.58	1.17	1.57	−1.86	4.23	−0.46	0.29	1:2:1	0.7
Juice content	6b	0.0	100034495|F|0	7.63	−0.46	0.114	2.29	7.47	NA	NA	1:1	1.1
Acidity	6b	130.73	100040907|F|0	8.43	−0.0	0.0	0.07	2.55	−0.08	3.03	1:2:1	4.0
Soluble solids	5	311.68	100065152|F|0	5.2	0.17	0.94	0.19	1.37	−0.3	3.32	3:1	2.0
TSS/TTA	6b	100	100020025|F|0–100077391|F|0	7.74	0.18	0.35	−0.37	1.40	0.76	3.5	1:2:1	7.2
Technological index	4	116.15	100044015|F|0	5.7	0.1	5.7	NA	NA	NA	NA	1:1	0.04
Technological index	5	340.0	100034974|F|0–100104371|F|0	12.47	0.05	1.36	0.08	4.5	−0.11	7.37	1:1:1:1	1.7
Technological index	6b	34.0	100084924|F|0–100033965|F|0	8.26	0.05	1.73	0.1	5.47	0.11	4.69	1:1:1:1	3.4
Technological index	7	83.51	100036043|F|0	5.87	0.07	2.74	0.06	2.25	0.09	3.13	3:1	1.8
Technological index	8	73.53	100043537|F|0	5.97	−0.09	5.32	0.05	1.52	0.05	1.56	1:2:1	8.7
Fruit per box	8	32.25	100020211|F|0	10.90	−19.63	5.04	14.51	2.88	−11.2	1.62	3:1	9.7

*LG* linkage group, *cM* position of the QTL in centiMorgans, *LOD* LOD score, *p* effect of Murcott tangor parental, *q* effect of Pera sweet orange parental, *pq* effect of both parents, *Seg* QTL segregation, *R* ^2^ percentage of the explained phenotypic variance

For the fruit quality traits (TTA, TSS, TSS/TTA and TI), 8 QTLs were identified. One QTL for acidity was mapped in LG6b at 130.73 cM, with a 1:2:1 segregation pattern due to the additive effect from Pera and a dominance effect, which were responsible for 4% of the phenotypic variation. For the TSS, another single QTL was detected in LG5 at 311.68 cM, with an R^2^ value of 2% and 3:1 ratio, as all the genetic effects were significant. Considering the TSS/TTA ratio, a unique region was mapped in LG6b at 100 cM, with a 1:2:1 segregation pattern due to the effect of Pera and a dominance effect, and this QTL explained 7.2% of the phenotypic variation. For the TI, five QTLs were identified: LG4 at 116.15 cM, LG5 at 340 cM, LG6b at 34 cM, LG7 at 83.51 cM and LG8 at 73.53 cM. The QTL located in LG4 had an LOD score of 5.7 and the lowest R^2^ observed for all traits, explaining 0.04%. This QTL showed only one significant additive effect from Murcott, segregating in a 1∶1 ratio. The second QTL mapped in LG5 had the highest peak for the TI, with an LOD score of 12.47 and an R^2^ of 1.7%; this QTL had experienced an additive effect from both parents, and a dominance effect was also detected, with the QTL segregating in a 1∶1:1:1 fashion. The QTL mapped in LG6b had an LOD score of 8.26 and an R^2^ of 3.4, with additive effects from its Murcott and Pera parents and a significant dominance effect that resulted in a 1∶1:1:1 segregation pattern. The only QTL detected in LG7 had an LOD score of 5.87 and an R^2^ of 1.8%, while segregating in a 3:1 fashion. The QTL detected for TI was identified in LG8 with an LOD score of 5.97, an R^2^ of 8.7, and a 1:2:1 segregation pattern.

The TI is derived from the following formula: TI = the juice content (%) x the TSS (Brix) x the weight of the citrus industry standard box (40.8 kg)/10,000. However, a direct comparison between these traits was not possible, as close QTLs were not found. For example, in LG5, a QTL was identified for the TSS and for the TI, but they were spaced 28.27 cM apart. This also occurred in LG6b, in which the QTLs were spaced 34 cM apart.

Some comparisons were made for all QTLs detected in present study (Tables 3 and 4). The correlation between ease of peeling and juice content was−0.19, which was also reflected in QTL mapping: e.g., in LG3, the QTLs for the detected traits were separated by 4.78 cM only. The genetic effects from both parents opposed one another, suggesting that the selection for the same direction of these traits is challenging due to their linkage and/or pleiotropic effect. Another comparison is between the number of seeds and diameter (correlation of−0.11), both of which were found in LG6b, at a distance of 2.25 cM, with the same segregation pattern (1:2:1), but with varying significant effects. In this case, the only genetic effect that was present for both traits were the additivity from Pera and the repulsion phase. For Murcott, the additive effect was significant only for the diameter, as the dominance was present only for the number of seeds. If the number of segments and TSS were considered (correlation of −0.09), QTLs were found for both traits in LG7 spaced by 5.44 cM. In this case, all genetic effects were significant; for Murcott tangor, the dominance was in coupling, and for the Pera, the effect was in repulsion.

In summary, QTL mapping detected one or two QTLs for all traits except the TI (five QTLs). In addition, the estimated proportions of the phenotypic variance (R^2^) explained by the mapped QTLs in this study were small. The highest R^2^ was 9.7. Budahn et al. [43] reported that major QTL effects are responsible for more than 45% of the phenotypic variance, which was not observed here. All these results clearly indicate that these traits are controlled by many genes and that the individual effect of one of these genes on the phenotype is small. These results illustrate the complexity of the characteristics associated with the production and/or quality of the fruits, but common regions were found for different traits: LG5 (311.68–315.12 cM), LG6b (32.25 cM), and LG 7 (311–315.12 cM). Those correlations were not strong but were still significant, which could suggest candidate regions for future studies to gain a better understanding of these traits.

Notably, comparison of the QTLs mapped in this study with those in previous studies is difficult because most of traits mapped here have not been investigated previously, and the map obtained in this study is the first to be integrated with DArTseq markers. Other factors that make the comparison of mapping results challenging are the different types of population and methodologies employed in the studies. For example, Sivieiro et al. [44] detected one QTL associated with the fruit number and one for seed production in the F_1_ progeny obtained from a C*itrus sunki* vs. *Poncirus trifoliata* cross using the pseudo-testcross strategy. García et al. [9] constructed genetic maps with isozymes, RFLPs, RAPDs and SSRs markers for *Citrus volkameriana* and *Poncirus trifoliata* by analysing an 80-tree progeny derived from its cross and investigated the linkages between these molecular markers and quantitative traits related to yield (fruit number, fruit weight and fruit size) and to fruit quality (seed number). They found three putative QTLs involved in the number of seeds per fruit. None of these papers reported phenotypic variance (R^2^) explained by the mapped QTLs [44].

Our approach was different because the map was obtained using the DArTseq markers in an integrated strategy. The model for mapping QTLs used here is the same as that adopted by Souza et al. [45] in a study of the genetic architecture of rubber tree traits related to growth under two conditions (winter and summer) using an integrated map. The phenotypic variation (R^2^) explained by the detected QTLs ranged from 2.72 to 8.97% in that study. The R^2^ in our study was quite similar because our values varied from 0.04% to 9.7%. These results clearly reflect that traits related to growth, development, production and fruit quality are complex and controlled by many genes with small effects on the phenotypic variation.

### Comparison of the linkage groups built from the *Citrus sinensis* genome

**Fig. 4:**
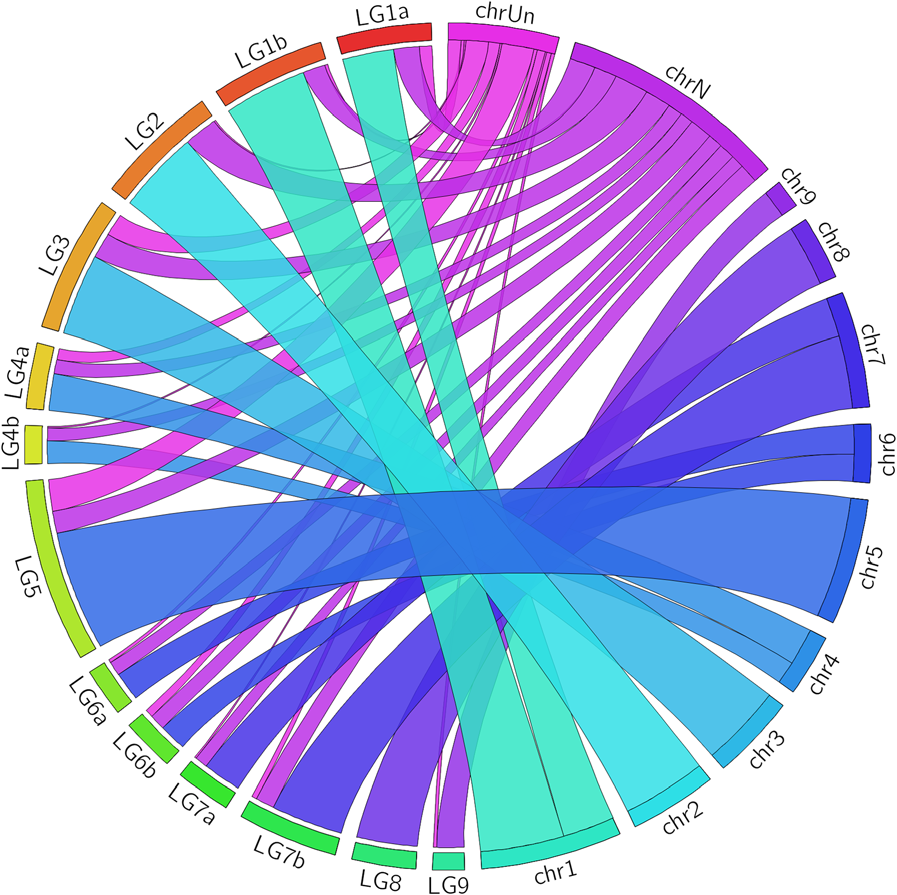
Comparison of the linkage groups (LGs) with the genome of *C. sinensis*, available at: http://citrus.hzau.edu.cn/orange/. Positioned on the left are the LG acronyms representing the constructed LGs (LG1a, LG1b, LG2, LG3, LG4a, LG4b, LG5, LG6a, LG6b, LG7a, LG7b, LG8, and LG9), which represent the integrated map of the “Murcott” tangor and the sweet *orange*. Previous Chr abbreviations (*Chr 1*, *Chr 2*, *Chr 3*, *Chr 4*, *Chr 5*, *Chr 6*, *Chr 7*, *Chr 8*, and *Chr 9*), illustrate the chromosomes of the reference genome used. Chr Un (*Chr* unassigned) is a segment of reference genome for all the sequences that were not placed on pseudo-chromosomes. Chr N represents all sequences that were not aligned in the *C. sinensis* genome

This approach was possible due to the existence of reference genome sequence available or when common markers exist between the different linkage maps [46]. Figure 4 shows the BLASTN results, in which essentially all the LGs were found to have full synteny with the reference genome used, except for markers that were unassigned to a chromosome (chromosome Un - Chr unassigned) or those that were not present in the genome of *Citrus sinensis* (Chr N).

Among the sequences, 69.30% of the marker sequences were located on the assembled chromosomes; 20.73% were not found in the reference genome used; and 9.97% were located on Chr Un, which reveals that the obtained linkage map contained sequences that were not previously positioned in the reference genome.

A comprehensive analysis of the draft genome of sweet orange (*C. sinensis*) [21] revealed 87.3% coverage of the estimated orange genome. Seventy-five percent of the assembled genome sequences were anchored in the nine LGs with the corresponding genetic markers used and 25% were unassigned in the nine LGs. Here we were able to determine the position of some of sequences among the 9 groups constructed for Murcott x Pera, that were previously unassigned in the assembly of the sweet orange genome. We believe that results may contribute to the assembly of the reference genome.

**Fig. 5:**
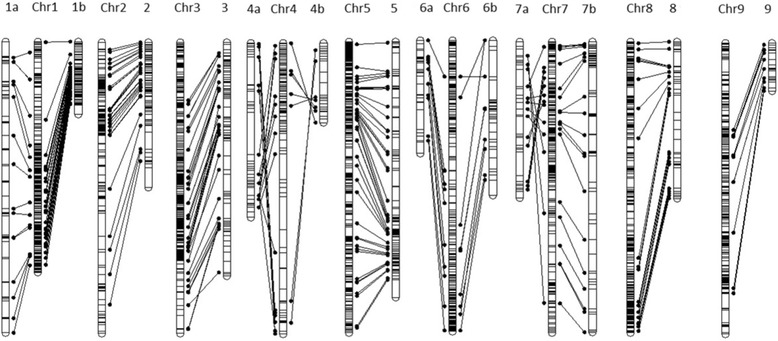
Comparison between the positions of the markers arranged on the linkage groups (LGs) of the integrated map and on the pseudo-chromosomes of *C. sinensis* (*Chr*). The LGs in this study are represented by 1a, 1b, 2, 3, 4a, 4b, 5, 6a, 6b, 7a, 7b, 8 and 9. *Chr 1*, *Chr* 2, *Chr* 3, *Chr* 4, *Chr* 5, *Chr* 6, *Chr* 7, *Chr* 8, and *Chr* 9 represent the chromosomes of the *C. sinensis* genome. The horizontal lines linking the groups and chromosomes represent the ordering and collinearity of the markers anchored on the map with the sequences of the genome

Once the map was identified as syntenic with the genome, the sequences present in the sweet orange genome were compared with the integrated linkage map constructed here (Fig. 5). The comparative analysis of the *C. sinensis* genome and the genetic map revealed significant co-linearity. However, despite the overall marker order being conserved between the developed map and the reference genome, intra-chromosomal rearrangements were evident, which can be explained by the existence of inversions and translocations. LG1, LG2, LG8 and LG9 had complete co-linearity with the reference genome. In the other groups LG3, LG4, LG5, LG6 and LG7 inversions and a change in the order of the markers were present. In linkage groups LG3, LG4, LG5, LG6 and LG7 inversions and a change in the order of the markers were present, and it could be explained as an error in marker order calculation or error in genome assembly or even possibly due to differences in chromosome rearrangements between the LG and the genome assembly.

### Gene content in the intervals between the detected QTLs

Overall, 19 QTL regions were analyzed for their gene content, for a total of 17 gene models. On average, one model was obtained for each detected QTL. In general, the predicted gene functions were related to different biological pathways (Table 5). Therefore, the roles of these genes in the traits found in this work need to be further functionally investigated.

**Table 5:** QTLs detected for each trait and predicted genetic models observed in the corresponding genomic intervals in the current version of the reference genome of *Citrus sinensis*

Trait	LG	Gene	Function
Height	4b	Cs4g03340	Mitochondrial outer membrane protein porin of 36 kDa
Height	5	orange1.1 t01158	HAT family dimerization domain-containing protein, expressed; Putative AC transposase; Putative AC9 transposase
Diameter	8	Cs8g03420	Branched-chain-amino-acid aminotransferase 2, chloroplastic
A/D	4b	Cs4g03340	Mitochondrial outer membrane protein porin of 36 kDa
Segments per fruit	5	orange1.1 t00827	Putative uncharacterized protein
Number of seeds	3	Cs3g07490	DNA polymerase epsilon, catalytic subunit A
Number of seeds	8	Cs8g02790	Actin-depolymerizing factor 5; Actin-depolymerizing factor (Fragment); Putative actin-depolymerizing factor 8; Actophorin; Cofilin; Cofilin-1B; Cofilin-1A; Cofilin/actin-depolymerizing factor homolog; Cofilin-4; Destrin
Ease of peeling	3	Cs3g07400	Transposable element protein, putative, Retrotrans_gag
Juice content	3		
Juice content	6b	Cs6g02380	Serine carboxypeptidase-like 29; Virulence-related protein Nf314; Similar to *Hordeum vulgare* carboxypeptidase D
Acidity	8	Cs6g07720	Mitogen-activated protein kinase-binding protein 1; WD repeat-containing protein 62; Echinoderm microtubule-associated protein-like 5; Novel protein similar to vertebrate mouse mitogen-activated protein kinase binding protein 1-like (MAPKBP1) (Fragment)
Soluble Solids	5		
TSS/TTA	6b	Cs6g07610	Putative acetyl co-enzyme A carboxylase carboxyltransferase alpha subunit; Acetyl-coenzyme A carboxylase carboxyl transferase subunit alpha, chloroplastic; Acetyl-coenzyme A carboxylase carboxyl transferase subunit alpha
TI	4	Cs4g03200	Probable xyloglucan endotransglucosylase/hydrolase protein 23; Xyloglucan endotransglucosylase/hydrolase protein 22; Brassinosteroid-regulated protein BRU1
TI	5	Cs5g14460	Coiled-coil domain-containing protein 94 homolog; Coiled-coil domain-containing protein 94; Cell cycle control protein cwf16; Protein CWC16; Synaptic vesicle transporter SVOP and related transporters (Major facilitator superfamily) (ISS)
TI	6b	Cs6g01810	Putative uncharacterized protein
TI	8	Cs7g05440	Lectin protein kinase family protein, putative, expressed; G-type lectin S-receptor-like serine/threonine-protein kinase SD2-5; Probable receptor-like protein kinase At5g20050
TI	8		
Fruits per box	6b	Cs8g03420	Branched-chain-amino-acid aminotransferase 2, chloroplastic; Branched-chain-amino-acid aminotransferase 6; Putative branched-chain-amino-acid aminotransferase 7

The co-location of QTLs has been used to suggest possible candidate genes or to validate candidate genes indirectly, mainly in forest species such as *Pinus taeda* [47], *Pinus pinaster* [48] and *Picea glauca* [49]. However, this approach can point to many genes of unknown function [50]. Here, we have identified sequences in QTL regions associated with the TI that show high homology with the enzyme xyloglucan endotransglycosylase/hydrolase (XTH) (Table 5). This enzyme participates in hemicellulose modification during the ripening of strawberry fruit [51]. During strawberry fruit ripening, significant modifications to the cell wall structure occur that are associated with increasing solubility of the wall components, decreasing polymer sizes and fruit firmness. Thus, these physical characteristics could somehow be related to the TI related to orange fruit ripening, affecting the TSS and juice content determined in our work, and these genes could be good candidates for functional analysis aimed at citrus breeding.

Regarding the number of seeds, one sequence was found with homology to the DNA polymerase epsilon, catalytic subunit A. The effect of DNA polymerase epsilon, catalytic subunit A, on the seed number has not yet been described, although this DNA polymerase was studied during gametophyte and seed development [52]. In Arabidopsis, the catalytic subunit of this complex is encoded by two genes, AtPOL2a and AtPOL2b, whereas the second largest regulatory subunit AtDPB2 is present as a unique copy. The gene AtDPB2 influences nuclear divisions, both in the embryo and in the endosperm, and AtPOL2 allows mitosis to proceed and may affect the cell cycle mechanisms of transcriptional regulation.

Functional analysis should be performed for all genes found in the detected QTL regions. However, not all genes found here have an apparent direct relation with the studied traits.

## Conclusion

The use of DArTseq markers, as well as the mapping strategy used herein, enabled the construction of an integrated map of Murcott tangor and Pera sweet orange. The resulting map contained the exact haploid number of chromosomes of the species and exhibited high genomic coverage. The map also presented a high degree of synteny and co-linearity with the *C. sinensis* genome. For the studied fruit quality traits, 19 QTLs were identified, but no QTLs were identified for fruit weight or peel thickness. The co-localization of QTLs at the noted intervals suggests the existence of genomic regions that are possibly related to the analyzed characteristics. This approach has helped in identifying candidate genes responsible for quantitative traits related to citrus fruit quality.

## Additional file

Additional file 1: Table S1. DArTseq dataset used for BLASTN analysis to compare the integrated linkage map constructed with the *Citrus sinensis* reference genome. (XLSX 38 kb)

## Abbreviations

AFLP: Amplified fragment length polymorphism
CIM: Composite interval mapping
DArT: Diversity arrays technology
fAFLP: Fluorescent amplified fragment length polymorphism
FCOJ: Frozen concentrated orange juice
LOD: Logarithm of odds
NFC: Not from concentrate juice
QTL: Quantitative trait loci
RAPD: Random amplified polymorphic DNA
RCD: Rapid chain delineation
RFLP: Restriction fragment length polymorphism
SNP: Single nucleotide polymorphism
SSR: Simple sequence repeat
TI: Technological index
TSS: Total soluble solids
TTA: Titratable acidity
